# Is bronchial thermoplasty safe in allergic bronchopulmonary aspergillosis or severe asthma with fungal sensitization?

**DOI:** 10.1186/s12890-021-01535-1

**Published:** 2021-05-18

**Authors:** Valliappan Muthu, Ritesh Agarwal

**Affiliations:** Department of Pulmonary Medicine, Postgraduate Institute of Medical Education and Research (PGIMER), Chandigarh, 160012 India

**Keywords:** Asthma, Cystic fibrosis, Abpm, Allergic bronchopulmonary mycosis, Aspergillosis, Aspergillus

We read with interest the case presented by Sasada et al., describing the occurrence of aspergillosis in a patient who underwent bronchial thermoplasty (BT) [1]. The isolation of *Aspergillus fumigatus*, the presence of elevated serum total IgE, and possible eosinophilic inflammation (high FeNO) suggest allergic sensitization to *A.fumigatus*. However, the diagnosis remains unproven as specific IgE or skin test against *A.fumigatus* was not performed in the index case. The authors mention that there was a remarkable improvement after BT. With only two sessions of BT being completed, the observed improvement is likely the effect of itraconazole therapy. Itraconazole offers excellent results in patients with severe asthma with fungal sensitization (SAFS) and allergic bronchopulmonary aspergillosis (ABPA) [2, 3]. The absence of bronchiectasis on computed tomography does not rule out ABPA and therefore, immunological investigations are required [4]. The prevalence of *Aspergillus* sensitization is about 28% in asthmatics and maybe as high as 50% in those with severe asthma [5, 6].

There is a significant burden of fungal allergy in Japan. In a recent Japanese study of 124 subjects with SAFS, 29% had allergic sensitization to at least one fungus, and sensitization to *A.fumigatus* was seen in 11% [7]. With such a high prevalence of fungal allergy in Japan, it would be reasonable to exclude SAFS or ABPA before BT. BT is a valuable addition in severe asthma management, especially for those who are not candidates for oral glucocorticoids or biologic therapy. Nevertheless, a thorough evaluation of coexisting conditions or complications is an indispensable component while evaluating severe asthma [8, 9]. Further, the trials evaluating BT or biologics in asthma have not included subjects with SAFS or ABPA. Hence, extrapolating the results beyond the trial population may be problematic. Thus, the index case should serve as a reminder to systematically evaluate severe asthma before contemplating treatment with newer modalities such as BT.

## Data Availability

Not applicable.

## Abbreviations

ABPA: Allergic bronchopulmonary aspergillosis
BT: Bronchial thermoplasty
SAFS: Severe asthma with fungal sensitization
